# An adaptive multi-graph neural network with multimodal feature fusion learning for MDD detection

**DOI:** 10.1038/s41598-024-79981-0

**Published:** 2024-11-18

**Authors:** Tao Xing, Yutao Dou, Xianliang Chen, Jiansong Zhou, Xiaolan Xie, Shaoliang Peng

**Affiliations:** 1 College of Computer Science and Engineering, Guilin University of Technology, Guilin, 541006 China; 2 College of Computer Science and Electronic Engineering, Hunan University, Changsha, 410082 China; 3 Hunan Key Laboratory of Psychiatry and Mental Health, Department of Psychiatry, National Clinical Research Center for Mental Disorders, National Center for Mental Disorders, National Technology Institute on Mental Disorders, The Second Xiangya Hospital of Central South University, Changsha, 410011 China

**Keywords:** MDD detection, GCN, Multimodal, Computer science, Scientific data

## Abstract

Major Depressive Disorder (MDD) is an affective disorder that can lead to persistent sadness and a decline in the quality of life, increasing the risk of suicide. Utilizing multimodal data such as electroencephalograms and patient interview audios can facilitate the timely detection of MDD. However, existing depression detection methods either consider only a single modality or do not fully account for the differences and similarities between modalities in multimodal approaches, potentially overlooking the latent information inherent in various modal data. To address these challenges, we propose EMO-GCN, a multimodal depression detection method based on an adaptive multi-graph neural network. By employing graph-based methods to model data from various modalities and extracting features from them, the potential correlations between modalities are uncovered. The model’s performance on the MODMA dataset is outstanding, achieving an accuracy (ACC) of 96.30%. Ablation studies further confirm the effectiveness of the model’s individual components.The experimental results of EMO-GCN demonstrate the application prospects of graph-based multimodal analysis in the field of mental health, offering new perspectives for future research.

## Introduction

MDD is a severe mental illness^1^. Patients with MDD often experience a lack of energy, loss of interest in life, and low mood. In severe cases, delusions or symptoms of hallucinations and auditory hallucinations may occur^2^. Severe depression negatively impacts daily life, work, sleep, diet, and physical health. Among adults with depression, 2-8% die by suicide^3^. Moreover, studies^4^ indicate that in high-income countries, 70-80% of individuals who die by suicide have a mental illness, with depression being the most common cause. In low- and middle-income countries, this proportion is about half. In recent years, the global prevalence of MDD has increased by 28%, with an actual count of 246 million cases^5^. Many studies^6^ show that timely therapeutic intervention can help alleviate the worsening of MDD.Therefore, there is an urgent need for an efficient and reliable method for detecting MDD.Currently, the clinical diagnosis of MDD primarily relies on psychiatric interviews and various depression rating scales to measure the severity of depression^5^, such as Patient Health Questionnaire (PHQ-9).However, the diagnosis of MDD primarily relies on patient self-reported symptoms and the interpretations of clinicians, and variations in MDD across individuals^7^ may lead to misjudgments about the patient’s condition.

Existing studies^8^^9^ indicates that MDD patients often speak slowly, with numerous pauses, and their content tends to be negative and lacking in energy^8^^9^. It has also been found that the electroencephalogram (EEG) waveforms of MDD patients show significant differences from those of healthy individuals^10^, highlighting the potential of EEG and voice analysis in the field of depression detection. To improve the effectiveness of MDD detection, many studies now utilize machine learning or deep learning to identify depression in patients. The inherent complexity of physiological signals poses a significant challenge for traditional machine learning methods^11^^12^, which often rely on manually extracted features. The crucial question is whether these handcrafted features possess sufficient discriminative power to enable traditional machine learning algorithms, such as Support Vector Machines (SVM), to effectively differentiate between different categories of individuals.

Current deep learning methods have shown certain limitations in extracting latent information from data. For instance, in studies dealing with EEG data, a common practice is to select only a few specific channels for analysis^13^, which may lead to overlooking important information contained in other channels, thereby affecting the comprehensiveness and accuracy of the final analysis. On the other hand, when using audio data as input, some studies tend to adopt Long Short-Term Memory networks (LSTM)^14^^15^ or Convolutional Neural Networks (CNN)^16^ as the primary architecture. Although these models perform well in handling time-series data and extracting local features, they may not fully mine and utilize global information and deeper features in audio data. This localized approach to information processing can limit the overall performance of the model. Therefore, to enhance the capabilities of deep learning models in the field of MDD detection, it is necessary to develop more advanced techniques and methods to more comprehensively and deeply analyze and utilize the information within these complex physiological data.

To tackle the challenges mentioned above, this article introduces a new multimodal graph neural network method for depression detection, named EMO-GCN. This method designs a set of graph neural networks for each modality to model the relationships within the data. By fusing features extracted from each modality into a multimodal embedding feature and introducing an attention mechanism on this embedding, the model focuses on the most important features in each modality. Finally, the attention-featured embedding is input into a classification network for subsequent MDD detection tasks. The main contributions of this article are as follows:

1. We propose a multimodal depression detection framework called EMO-GCN, which uses multiple graph convolutional networks to extract structural features from EEG signals and acoustic features from speech, achieving effective multimodal feature fusion. These fused multimodal representations provide more accurate indicators for depression detection, thereby significantly enhancing performance.
2. We propose a Multi-GCN module that combines stacked graph convolution and graph pooling layers, introducing a structural learning mechanism that reconstructs the graph structure through sparse attention after pooling. This approach accurately captures complex graph features while preserving node relationships, enabling efficient representation of data characteristics.
3. We evaluate our proposed method on the MODMA public dataset through extensive experiments. The results demonstrate that our approach outperforms existing baseline algorithms in the depression detection task, achieving an accuracy of 96.30%, with its effectiveness further validated through comprehensive ablation studies.

The rest of this paper is structured as follows. “Related work” briefly discusses related work and technologies in the task of MDD detection. In “Methodology”, we provide detailed information about the method we propose. After that, “Experiments setup” introduces our experimental design and settings and describes the datasets. In “Results”, findings from comparison experiments with other models and the ablation study are presented. Then, in “Discussion”, we discuss the current limitations of our work and propose some possible future directions. Finally, “Conclusion” concludes the paper.

## Related work

### Traditional depression detection

In the field of clinical depression diagnosis, there are already many detection methods. The most common methods involve psychological tests or questionnaires. For example, the PHQ-9 is a commonly used screening tool for depression^17^. The PHQ-9 contains 9 questions, mainly asking about the frequency of depressive symptoms over the past two weeks and can be completed in about 5 minutes. Another commonly used clinical assessment scale is the HAMD^18^. It is a clinician-administeblack tool consisting of 17 items to assess the severity of depressive symptoms, with scores ranging from 0 to 52. The higher the score, the more severe the depression. The drawback of traditional questionnaire methods is their subjectivity and variability^19^.Additionally, these scales have limitations^20^. When using self-assessment scales like PHQ-9, there can be differences in understanding among participants, which may lead to varying scores. With other-assessment scales like HAMD, different doctors may give inconsistent scores for the same patient. Therefore, it’s challenging to accurately detect whether a subject has depression solely through the use of scales.

To objectively diagnose depression, machine learning is increasingly being used to identify depression. For example, Deshpande et al.^21^ analyzed tweets collected using Naive Bayes and SVM methods, identifying potential depressive moods among a large set of user data. However, significant noise in the collected tweets before preprocessing, such as third-person references and news quotes, led to the elimination of about one-third of the data. Islam et al.^22^ utilized SVM, Decision Tree, KNN, and other methods to build predictive models for identifying and processing emotion data related to depression in Facebook posts. Despite the use of various machine learning techniques, the accuracy rates were only between 60$\%$ to 80$\%$, indicating room for improvement.

### Graph neural network detection method

Graph Neural Network(GNN) was first introduced by Scarselli et al.^23^, defining them as a type of recursive neural network capable of directly operating on graph-structured data. GNN are gradually gaining traction in the medical field as well. Zhao et al.^24^ proposed ECGNN, composed of a feature extractor backbone and a GNN module, which extracts electrocardiogram (ECG) features for the diagnosis of cardiovascular diseases. Wang et al.^25^ introduced the MGREL, integrating knowledge extraction and graph learning channels. This model uses graph representation learning to acquire network topology representations for predicting associations between genes and diseases. The study by Fritz et al.^26^ combines GNN with epidemiological models to enhance the predictive accuracy of weekly COVID-19 cases across various regions in Germany.

GNN have also been applied in depression detection. Yu et al.^27^ proposed a method based on GNN that combines temporal and spatial features of functional near-infrared spectroscopy (fNIRS) data for automatic depression recognition. Sun et al.^28^ constructed two GNN modules sequentially to explore latent connections within and between audio signals, providing relevant cues for detecting depression in the model. Luo et al.^29^ designed a Graph Convolutional Gated Recurrent Unit (GCGRU) module to capture the temporal dynamic changes in brain networks within EEG data, further extracting differential features between depressed individuals and healthy controls.

### Multimodal depression detection

With the advancement of computer technology, depression detection methods have evolved from unimodal to multimodal approaches, particularly combining speech and EEG data to capture a more comprehensive set of multidimensional features in individuals with depression. Qayyum et al.^13^ integrated different levels of speech and EEG features and applied visual transformers and various pre-trained networks, significantly improving the diagnostic effectiveness for patients with depression. Zheng et al.^30^ proposed a novel time-convolutional transformer with knowledge embedding to link audio and EEG, capturing effective features and enhancing the performance of depression detection models. Addressing both heterogeneity and homogeneity between the speech and EEG modalities, Chen et al.^31^ proposed a multimodal fusion strategy based on graph neural networks to explore potential relationships between samples. Current research indicates that multimodal methods demonstrate clear advantages in depression detection, as integrating data from different modalities, such as audio and EEG, enables a more comprehensive reflection of the emotional and behavioral characteristics of individuals with depression. Although some progress has been made with multimodal depression detection methods, there remains a limited number of methods in this area, particularly those based on EEG and speech. Existing approaches also have certain limitations. For example, Chen et al.’s^31^ method introduces a reconstruction network, which increases model complexity and computational burden and poses a higher risk of overfitting when data samples are limited. In Qayyum et al.’s^13^ method, EEG and speech data undergo numerous processing and feature extraction steps, including spectrogram generation and temporal correlation extraction; while these steps help improve classification accuracy, they also increase computational complexity.

## Methodology

**Figure 1:**
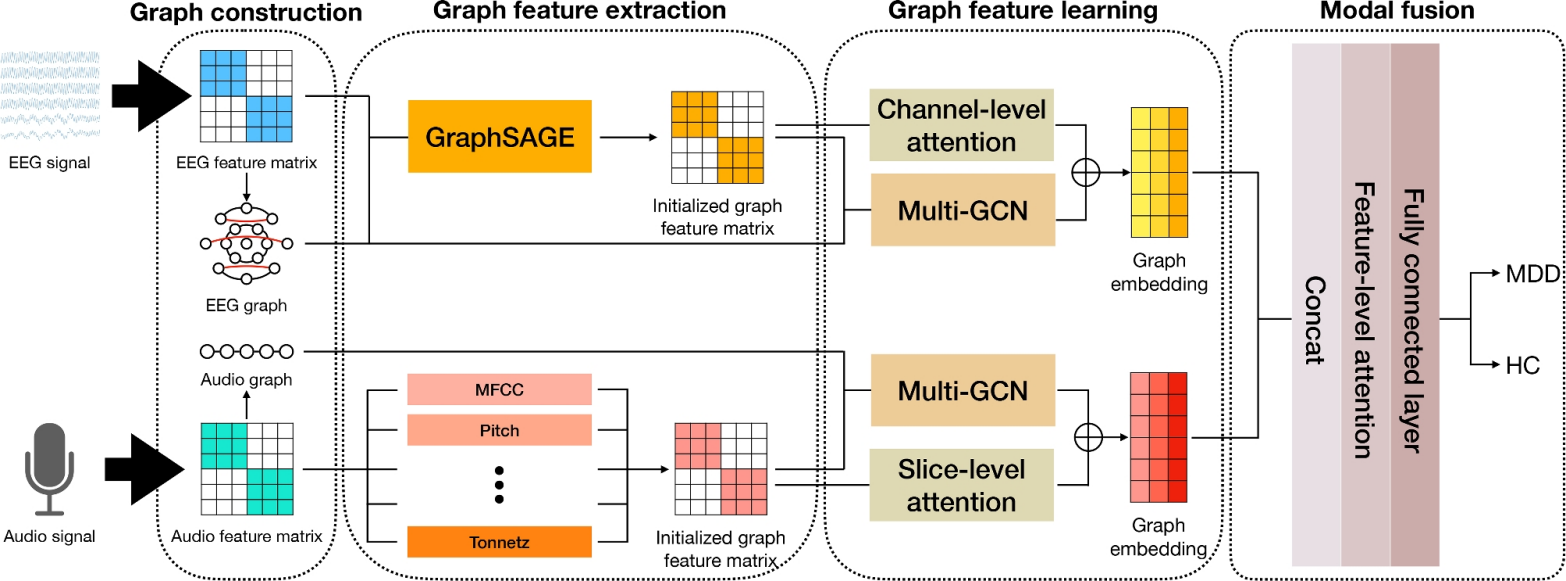
Framework of EMO-GCN.

In this section, we will introduce EMO-GCN, which consists of a graph feature vector extraction module for each modality and a graph neural network module.Figure1 shows the framework of EMO-GCN. The graph feature vector extraction module takes EEG and audio data from patients as inputs, with the data undergoing preprocessing and feature extraction to produce initial graph feature matrices for each modality. The graph neural network module learns from these initial graph feature matrices, generating new subgraphs through iterative execution of graph convolution, graph pooling, and structural learning mechanisms. Node features within the subgraphs are aggregated to produce fixed-size graph embedding vectors. Finally, the graph embedding vectors from both modalities are fused and an attention mechanism is introduced before feeding them into a fully connected layer to obtain the final detection results.

### Problem definition

Given a multimodal dataset $\{D_i\}^N_{i=1}$, comprising *N* multimodal patient samples, $\{D_i\}^N_{i=1}$ includes audio data $M^a \in R^{N \times Row^a \times Col^a}$ and EEG data $M^e \in R^{N \times Row^e \times Col^e}$, along with one-hot labels for each sample $Y \in R^{N\times C}$. Here, ${Row}^a$ and ${Row}^e$ respectively represent the number of sample points in the audio data and the number of electrodes in the EEG data, where the sample points equal the audio duration multiplied by the sampling rate; $Col^a$ and $Col^e$ respectively represent the feature dimensions of $M^a$ and $M^e$; and *C* represents the number of label categories. In this article, our objective is to input the multimodal dataset $\{D_i\}^N_{i=1}$ into the model, and subsequently output the detection results for each sample ${\hat{Y}}_i \in R^{1\times C} (i=1,2,3,\cdots )$.

For each sample in $M^e$, obtained using an EEG device with $n_e$ electrodes, we construct an arbitrary graph $G^{e}=( \nu ^e, \varepsilon ^e, X^e)$, where $\nu ^e$, $\varepsilon ^e$, and $X^e \in R^{n_e \times m_e}$ respectively represent the nodes, edges, and feature matrix of $G^e$, with $m_e$ denoting the dimension of $X^e$. Also, let $A^e \in R^{n_e\times n_e}$ be the adjacency matrix representing the graph connectivity information, where the element $A^e(j,k)$ indicates the connection status between the *j*-th and *k*-th electrodes. If these electrodes are connected, then $A^e(j,k)=1$; otherwise, $A^e(j,k)=0$. Similarly, for each sample in $M^a$, consisting of $n_a$ audio segments, we construct a graph $G^a=(\nu ^a ,\varepsilon ^a, X^a)$, where $\nu ^a$, $\varepsilon ^a$, $X^a \in R^{n_a \times m_a}$ respectively represent the nodes, edges, and feature matrix in $G^a$. The connectivity information of the nodes in $G^a$ is stored in the adjacency matrix $A^a \in R^{n_a \times n_a}$.

### Graph construction

In this subsection, we describe the process of constructing graph structures for $M^e$ and $M^a$.

**Figure 2:**
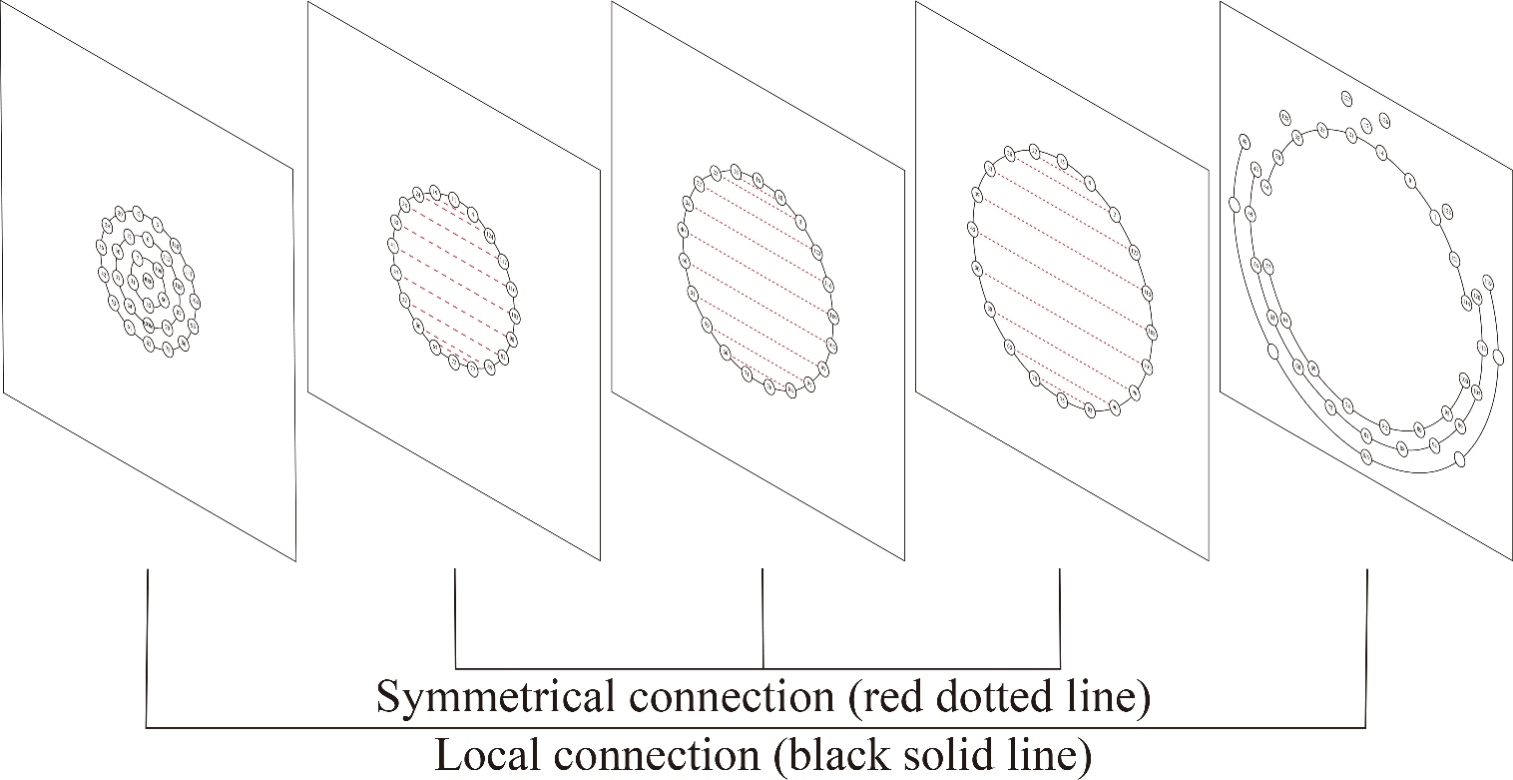
Local connection and symmetrical connection.The red dotted line represents symmetrical connections, while the black solid line represents local connections

The spatial distribution of electrodes in the EEG collection device can help us preliminarily construct the graph structure of the EEG. This allows for accurate modeling of local activities in various brain regions. By considering each electrode as a node in $G^e$ and connecting corresponding nodes based on the spatial distribution of electrodes, we can obtain the initial adjacency matrix $A^e_{init}$. Additionally, a study focusing on the EEG symmetry in patients with depression found that analyzing the EEG symmetry of homologous regions in the left and right brain is significant for the diagnosis of depression^32^. Inspired by this, we empirically selected some symmetric electrodes from the left and right hemispheres and connected them, resulting in a symmetrically distributed electrode adjacency matrix $A^e_{sym}$. The final adjacency matrix $A^e$ for $M^e$ is derived using the formula as $A^e=A^e_{init}+A^e_{sym}$.Figure 2 shows the graph structure of EEG data. The resulting adjacency matrix $A^e$ can express the connectivity between brain localities and between the left and right hemispheres. Such an adjacency matrix not only helps in exploring the associations between local regions of the brain but also assists the graph neural network module in learning and recognizing cross-hemispheric brain activity patterns. It can provide a more comprehensive representation of brain activity data, which is beneficial for training a more accurate model.

The temporal nature of $M^a$ is key to constructing its graph structure.The audio samples are cut into slices of equal time length, and each slice is regarded as a node. The adjacency matrix $A^a \in R^{n_a\times n_a}$ for $M^a$ can be obtained using the following formula:

1 $$\begin{aligned} A^a(j,k)={\left\{ \begin{array}{ll} 1 & \text {if } |j - k| = 1 \\ 0 & \text {otherwise} \end{array}\right. } \end{aligned}$$

When $|j - k| = 1$, it indicates that the *j*-th and *k*-th nodes are adjacent. In this case, we set the element in $A^a$ corresponding to the connection between these two nodes to 1, signifying that the adjacent nodes are connected. For other cases, the element in $A^a$ representing the connection between the *j*-th and *k*-th nodes is set to 0. This graph structure naturally reflects the temporal sequence of $M^a$ and is capable of capturing changes in the audio signal over different time periods. A sequentially connected graph structure can also assist in exploring the emotional changes of patients over a period of time. This is helpful for analyzing the temporal information in $M^a$ and learning complex audio features.

### Graph feature extraction module

The feature extraction module converts raw EEG signals and audio data into graph-level feature matrices. These matrices serve as the initial graph features for training and learning within the graph neural network. Since the two modalities have their own characteristics, we have designed suitable feature extraction modules for each modality separately.

Due to the high-dimensionality characteristic of $M^e$, reflected in the data containing a large number of electrode channels, each electrode collects a substantial amount of data, involving multi-dimensional time-series signal acquisition. Excessively high dimensions are not conducive to model fitting and also require more time for training. To preserve the complete electrode channels while addressing this challenge, we use the GraphSAGE algorithm to perform dimensionality reduction on the original $M^e$. This involves learning node embeddings by subsampling neighboring node features on the graph dimension. This approach effectively reduces the data dimensionality, decreasing the computational training load. Given an EEG signal data $M^e_i = \{e_1, e_2, \cdots , e_{n_e} \}$ with $n_e$ channels, and its graph structure $G^{e}=( \nu ^e, \varepsilon ^e)$, we first calculate the average feature of the neighboring nodes for each node. For each node *v* and its set of neighboring nodes *N*(*v*), the neighbor average feature $h_{N(v)}$ is computed as $h_{N(v)} = \text {mean}\{ e_u, \forall u \in N(v) \}$. After obtaining $h_{N(v)}$, we concatenate each node’s feature $X^e_v$ with the average feature of its neighbors to get $h_v$, which is given by $h_v=e_v \parallel h_{N(v)}$. Subsequently, $h_v$ is fed into a linear layer and an activation function is applied. Let $W$ and $b$ represent the weight and bias of the linear layer, respectively. The new feature representation $h'_v$ is obtained using the following formula:

2 $$\begin{aligned} h'_v=\text {ReLU}(W \cdot h_v + b) \end{aligned}$$

Finally, the $h'_v$ calculated for all nodes are combined to form a matrix, resulting in the initial graph feature matrix $X^e_0 \in R^{n_e\times m'_e}$ for the subsequent graph neural network. Here, $m'_e$ is the size of the feature vector after the feature extraction process. The initial graph feature matrix obtained through dimensionality reduction retain all channels, enabling the graph neural network to explore the associations between channels.

Although both $M^a$ and $M^e$ are waveform data, when processing $M^a$, it is crucial to focus on the patient’s emotional changes from a speech perspective. Features obtained from a perspective different from EEG can complement other aspects of MDD characteristics in a single modality. Assuming that each sample in $M^a$ has $n_a$ audio segments, we compute various sound features in each segment that are effective for emotion recognition, such as Mel Frequency Cepstral Coefficients (MFCC), pitch, Root Mean Square (RMS) energy, and Mel-spectrogram(Mel). Let the $j$-th feature of the $i$-th audio segment be denoted as $f_{ij}$, leading to an audio feature row $r_i = [f_{i1}, f_{i2}, \ldots , f_{im'_a}]$, where $m'_a$ is the size of the feature vector after extraction. Subsequently, we concatenate all the obtained audio feature rows along the Y-axis to form the feature matrix. The audio emotion feature matrix $X^a_0 \in R^{n_a \times m'_a}$, obtained through the calculation of audio emotional features, facilitates a deeper understanding of the relationship between speech and emotion in subsequent analyses.

### Multi-GCN component

**Figure 3:**
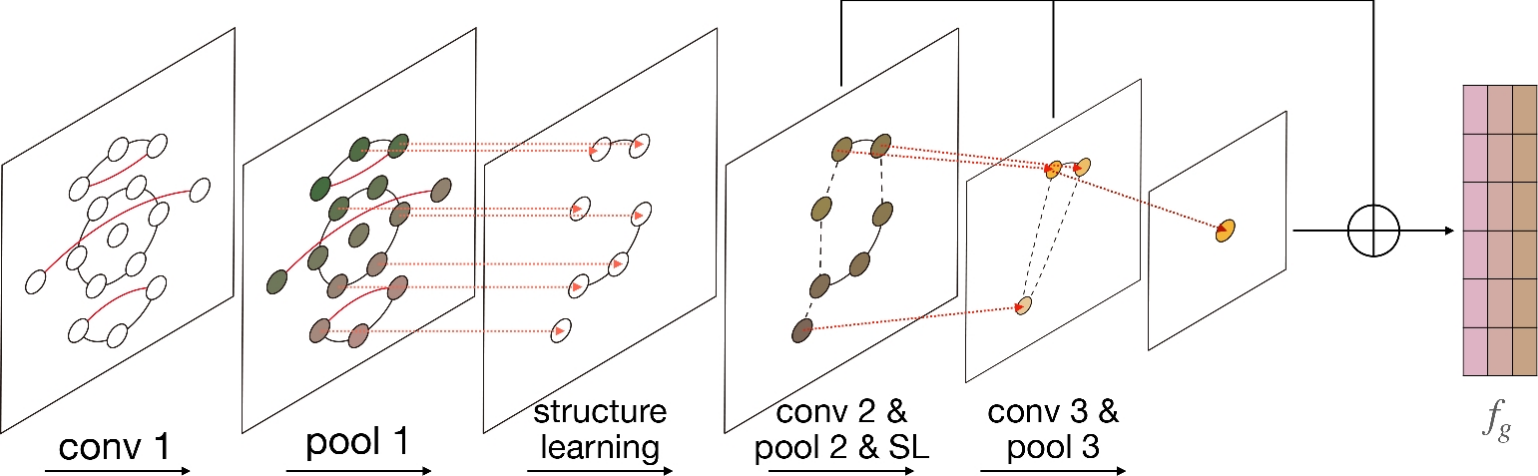
Workflow of multi-GCN component.

In the Multi-GCN component, a graph convolution layer and a graph pooling layer together form a basic unit. By stacking such units in three layers, we have constructed the complete Multi-GCN. Such a structure is capable of capturing more complex and abstract features of the graph structure. Figure 3 shows the workflow of Multi-GCN component.

#### Graph convolutional network

Graph Convolutional Network (GCN)^33^ is a powerful method for learning and capturing graph structures, which can effectively understand both local and global relationships within the graph to enhance prediction and classification accuracy. The initial graph feature matrix of $M^e$ and $M^a$ are obtained through the feature extraction module. If traditional LSTM or CNN methods are used to process the feature matrix, they fail to capture and learn the complex relationships and dependencies between nodes of $M^e$ and $M^a$ at the graph level. GCNs, on the other hand, have a unique advantage in scenarios that require capturing and utilizing complex relationships between nodes. The key idea of GCN is to update the feature representation of each node by aggregating the feature information of adjacent nodes. The basic operation of GCN can be represented by the following formula:

3 $$\begin{aligned} H^{(l+1)} = \sigma \left( {D}^{-\frac{1}{2}} {A} {D}^{-\frac{1}{2}} H^{(l)} W^{(l)} \right) \end{aligned}$$

Where $H^{(0)} = X^0, H^{(l)}$ is the node feature representation of the $l$-th layer, $H^{(l+1)}$ is the node feature representation of the $(l+1)$-th layer, $A$ is the adjacency matrix of the graph, $D$ is the diagonal matrix, $W^{(l)}$ is the weight matrix of the $l$-th layer, and $\sigma$ is the activation function. In this formula, $D^{-\frac{1}{2}}AD^{-\frac{1}{2}}$ is responsible for normalizing the adjacency matrix to maintain numerical stability when aggregating the features of neighboring nodes. $H^{(l)}W^{(l)}$ represents feature transformation, i.e., mapping the current features to a new feature space through the weight matrix. Finally, the activation function $\sigma$ provides a non-linear mapping, allowing the network to learn complex patterns.

#### Graph pooling

Graph structure data may contain noise and information irrelevant to MDD detection. We employ graph pooling to remove redundant information from the data. The purpose of graph pooling is to reduce the number of nodes in a graph neural network while retaining the most important information as much as possible. First, a score is assigned to each node, which is calculated using the following formula:

4 $$\begin{aligned} \text {score} = \left\| \left( I - D^{-1} A \right) H^{(i)} \right\| \end{aligned}$$

Where $I$ is the identity matrix, $D$ is the diagonal matrix, $D^{-1}$ is the inverse of $D$, and $H^{(i)}$ is the feature matrix of the nodes. This formula evaluates the information content of a node by calculating the difference between each node and its neighbors. This difference is computed through the expression $(I - D^{-1}A)H^{(i)}$, which essentially looks at the difference between the features of a node and those of its neighbors. After calculating the information score of each node, the nodes with the highest scores are selected according to the calculated scores, formula described as $\text {idx} = \text {top}(\text {score})$. Subsequently, the node feature matrix and adjacency matrix are updated based on the high-scoring nodes selected:

5 $$\begin{aligned} {\tilde{H}}^{(i+1)}&= H^{(i)}(\text {idx}, :) \nonumber \\ {\tilde{A}}^{(i+1)}&= A^{(i)}(\text {idx}, \text {idx}) \end{aligned}$$

$H^{(i)}(\text {idx},:)$ represents selecting the features corresponding to the highest scoring nodes from the original node feature matrix $H^{(i)}$. $A^{(i)}(\text {idx},\text {idx})$ indicates updating the adjacency matrix according to these nodes, meaning retaining the connections between these high-scoring nodes. Graph pooling is used to reduce the number of nodes and edges in $G^e$ and $G^a$. Identifying and preserving information that is effective for the MDD detection task can reduce the negative impact of irrelevant or misleading information on MDD detection performance.

#### Structure learning

In our framework, we constructed graph structures for $M^e$ and $M^a$ separately. However, graph pooling may lead to the disconnection of originally closely related nodes in the subgraph, thereby losing the integrity of the graph structure information and hindering the message passing process. To learn a refined graph structure after graph pooling, encoding the underlying pairwise relationships between nodes, we introduced a structure learning mechanism following the graph pooling operation. This mechanism learns a sparse graph structure through a sparse attention mechanism. For the subgraph $G^k_i$ obtained after the $k$-th layer pooling of graph $G_i$, we take its adjacency matrix information $A^k_i \in R^{n^k_i \times n^k_i}$ and node feature matrix $H^k_i \in R^{n^k_i \times d}$ as inputs. The structure learning mechanism is implemented through a single-layer neural network, parameterized by the weight vector $\textbf{a} \in R^{1 \times 2d}$. The similarity score between nodes $v_p$ and $v_q$ can be represented as follows:

6 $$\begin{aligned} E^k_i(p,q)= \sigma (\textbf{a}[H^k_i(p,:)\,||\,H^k_i(q,:)]^T) + \lambda \cdot A^k_i(p,q) \end{aligned}$$

In the above formula, $\sigma (\cdot )$ denotes the activation function. $H^k_i(p,:)$ and $H^k_i(q,:)$ represent the $p$-th and $q$-th rows of the matrix $H^k_i$, respectively, corresponding to nodes $v_p$ and $v_q$. When $A^k_i(p,q) > 0$, it indicates a direct connection between the two nodes, and the attention mechanism tends to assign higher similarity scores to directly connected nodes. At the same time, it attempts to learn the underlying pairwise relationships between directly connected nodes and nodes that are not directly connected, with $\lambda$ being the balancing parameter between the two. To make the similarity scores between different nodes more easily comparable, the structure learning mechanism uses the softmax function for normalization:

7 $$\begin{aligned} S^k_i(p, q) = \frac{\exp (E^k_i(p, q))}{\sum _{m=1}^{n^k_i} \exp (E^k_i(p, m))} \end{aligned}$$

The non-zero values resulting from the softmax transformation can lead to a densely connected graph, introducing a significant amount of noise. To address this issue, we use the sparsemax function, which converts the original softmax function into a piecewise function. This approach avoids introducing noise in the structure learning process:

8 $$\begin{aligned} S^k_i(p, q)&= \text {sparsemax}(E^k_i(p, q)) \nonumber \\ \text {sparsemax}(E^k_i(p, q))&= \left[ E^k_i(p, q) - \tau (E^k_i(p, :))\right] _+ \end{aligned}$$

In the above formula, $[x]_+ = \text {max}\{0,x\}$. The function $\tau (\cdot )$ determines the threshold for the sparsemax function transformation, thereby producing a sparse distribution. Through the aforementioned operations on the subgraphs, the model can learn a more refined subgraph structure. This ensures the integrity of the graph structure information after graph pooling operations, while not introducing additional noise. To generate a fixed-size graph-level embedding, we concatenate the results of mean pooling and max pooling for each subgraph, aggregating the representations of all nodes within the subgraph:

9 $$\begin{aligned} r^k_i = \frac{1}{\sigma } \left( \sum _{p=1}^{n^k_i} H^k_i(p,:) \, || \, \max _{q=1}^{d} H^k_i(:,q) \right) \end{aligned}$$

Then, we sum up the node representations from different subgraphs to obtain a graph neural network embedding $f_g = r^1_i + r^2_i + \ldots + r^K_i$.

In Multi-GCN, we alternated between graph convolutional networks and graph pooling. The graph convolutional network effectively extracts relational features between nodes by propagating information between the nodes in $G^e$ and $G^a$. Graph pooling reduces the complexity of the graph, thereby lowering the risk of overfitting and also minimizing noise in the data. Utilizing a structural learning mechanism after graph pooling can address the issue of lost node associations in subgraphs resulting from pooling.

### Modal fusion

For the initial graph feature vectors $X^e_0$ and $X^a_0$ outputted from the graph feature vector extraction module, we applied attention mechanisms at both the EEG channel-level and the audio slice-level to assign greater importance to electrode channels and time segments that exhibit characteristics of depression. The attention scores at the EEG channel-level and the audio slice-level are calculated using the following formulas:

10 $$\begin{aligned} \alpha&= \text {softmax}(V \tanh (WX_0 + b)) \nonumber \\ f_b&=\sum _{i=1}^{n}\alpha _iX_i,(n=n_e\ for\ EEG,n=n_a\ for\ audio) \end{aligned}$$

Where $V$, $W$, and $b$ are parameters at the EEG channel-level and audio slice-level learned during training, $\alpha$ represents the attention scores, and $f_b \in {\mathbb {R}}^m$ is a context vector that encodes the EEG signals of $n_e$ electrode channels or the audio signals of $n_a$ audio slices for different modalities. The context vectors $f_b$ from each modality are used for the final results.

We aggregate the representations of all nodes in the subgraphs generated by Multi-GCN from each modality and sum them up to obtain a fixed-size graph-level representation.

11 $$\begin{aligned} f_g = \sum _{i=1}^l\sigma \left( \frac{1}{n_i} \sum _{p=1}^{n_i} H^{(i)}(p,:) \parallel \max _{q=1}^d H^{(i)}(:,q)\right) \end{aligned}$$

$f_g \in {\mathbb {R}}^{2d}$ is the final aggregated feature vector, where $l$ represents the number of subgraphs, and the $\parallel$ operation denotes concatenating the results of mean pooling and max pooling for each subgraph. Subsequently, the two features $f_b$ and $f_g$ are concatenated to obtain the final graph embedding feature for each modality individually $f_{\text {embedding}} = f_b \parallel f_g$. For the $f_{\text {embedding}}$ of each modality, we first concatenate them and then apply a feature-level attention mechanism to assign greater importance to specific features indicative of depression.

12 $$\begin{aligned} \text {output}=\text {Att}_{\text {EEG}}(f_{\text {embedding}}^e)\parallel \text {Att}_{\text {Audio}}(f_{\text {embedding}}^a) \end{aligned}$$

Where $f_{\text {embedding}}^e$ and $f_{\text {embedding}}^a$ are the graph embedding features derived from the neural network for the EEG and audio modalities, respectively. The attention weighting function $\text {Att}_{\text {modality}}(\cdot )$ is defined as:

13 $$\begin{aligned} \text {Att}_{\text {modality}}(f_{\text {modality}}) = f_{\text {modality}} \odot \sigma (w_{\text {modality}}) \end{aligned}$$

In this context, $\odot$ denotes the Hadamard product, and $w_{\text {modality}}$ is learned through training. This attention mechanism allows the model to dynamically adjust the contributions of the two modalities when processing the fused features, thereby enhancing the flexibility and effectiveness of handling multimodal data.

## Experiments setup

### Dataset

The Multimodal Open Dataset for Mental Disorder Analysis (MODMA) offers clinically accurate data for analyzing mental disorders like depression. It includes data from patients professionally diagnosed with depression and matched controls. The dataset contains resting-state and stimulus-state EEG data from 51 subjects (22 with depression, 29 controls), recorded at 250 Hz using a 128-channel HydroCel Geodesic Sensor Net, with electrode impedance below 50 k$\Omega$. Audio data from the subjects were recorded using a Neumann TLM102 microphone and an RME FIREFACE UCX interface at 44.1 kHz and 24-bit depth, under environmental noise below 60 dB. Each patient has 29 audio segments. To match the number of samples between audio and EEG data, we divided the EEG data into 29 segments, resulting in 638 MDD samples and 841 healthy control (HC) samples. Table 1 presents the demographics of the dataset segmented across different dimensions.

**Table 1:** Patient demographic information (values are number of people)

Characteristic	Category	MDD	HC	Total
Gender	Male	16	20	36
	Female	6	9	15
Age (years)	< 45	19	27	46
	$\ge$ 45	3	2	5
Education (years)	$\le$ 12	12	3	15
	> 12	10	26	36
PHQ-9 (score)	$\le$ 19	13	29	42
	> 19	9	0	9

### Performance metrics

In the experiments of this paper, we use four key metrics to evaluate the performance of the model: accuracy (ACC), precision (PRE), recall (REC), and F1 score. These indicators measure model performance in terms of overall correctness, accuracy of positive class detection, completeness of positive class identification, and the balance between the two. Combining these four indicators, we can fairly evaluate and optimize model performance in different application scenarios.

### Implementation details

Data splitting: In our experiments, we used a k-fold cross-validation method (k=10) and averaged the 10 validation results to obtain the overall performance metric of the model, thereby validating the model across different partitions of the dataset to ensure robust generalization ability.

Parameter settings: In the experiments of this article, we used the MODMA dataset as the experimental data. We denote $M^e$ and $M^a$ as the 128-channel resting-state EEG data and audio data from the MODMA dataset, respectively. For the graph structures $G^e$ and $G^a$ corresponding to $M^e$ and $M^a$, we set their number of nodes to $n_e=128$ and $n_a=32$, respectively. The dimensions $m'_e$ and $m'_a$ of $X^e_0$ and $X^a_0$, obtained through the graph feature vector extraction module for the two modalities, are set to 600 and 580, respectively.

### Baselines

We present the results of 15 baseline methods for the depression detection task on the MODMA dataset, covering multimodal, EEG, and audio approaches. Additionally, we include two EMO-GCN variants: EMO-GCN-$\alpha$ (EEG only) and EMO-GCN-$\beta$ (audio only).

Multimodal models:

1. MS2-GNN^31^: offers an effective multimodal fusion approach based on graph neural networks that significantly enhances the precision in identifying depression.
2. Ahmed et al.^34^: proposed a multimodal classifier based on attention mechanisms that combines selective dropout and normalization techniques to handle missing modalities in different multimodal datasets.
3. EfficientNet^13^: is a variant of CNN models that scales model depth, width, and resolution in a balanced way, thereby optimizing performance and computational cost.
4. MobileNet^13^: is a lightweight CNN model specifically designed for mobile and embedded devices, optimized for speed and memory usage without significantly affecting accuracy.
5. Hu et al.^35^: used large language models (LLMs) to perform mental health assessments on multimodal data through zero-shot and few-shot prompting.

EEG models:

1. Tasci et al.^36^: introduced a novel and computationally light manual feature engineering technique called the Twin Pascal Triangle Layer Pattern (TPTLP).
2. SGP-SL^37^: progressively optimizes EEG-based graph structures by utilizing multiple self-attention graph pooling modules and introduces a soft label strategy to construct the loss function, enhancing the discriminability of features.
3. Soni et al.^38^: used the Node2vec algorithm to generate node embeddings of EEG data as features to distinguish between patients with depression and healthy subjects.
4. Shen et al.^39^: proposed an adaptive channel fusion method based on EEG signals, enhancing the separability of difficult samples by assigning higher weights to their losses through an improved focal loss (FL) function.
5. Sun et al.^40^: extracted various types of EEG features to comprehensively represent the EEG signals of MDD patients, using machine learning algorithms and statistical analysis to evaluate these EEG features.

Audio models:

1. GNN-SDA^41^: includes a GNN-based domain alignment module and an uncertainty-guided optimization module, which respectively achieve multi-domain alignment through an information propagation mechanism and analyze the uncertainty of pseudo-labels to mitigate the adverse effects of noisy predictions.
2. Gheorghe et al.^42^: performed audio preprocessing, multidimensional feature extraction, and classified samples using a multilayer perceptron (MLP) and a 1D-CNN.
3. Sun et al.^28^: constructed a GNN model that integrates the temporal sequence information within audio signals, the potential associations between different audio pieces, and the extraction of emotional features.
4. Chen et al.^43^: constructed a large database with 1,479 speech feature samples for modeling. Through 10-fold cross-validation and algorithm selection, they established a decision tree model for MDD screening.
5. Das et al.^44^: combined extracted MFCC and spectrogram features into multimodal data based on audio data, using a CNN model with optimized residual blocks and a “glorot uniform” kernel initializer to identify MDD patients.

## Results

In this section, we evaluate the effectiveness of the proposed EMO-GCN on the MODMA^45^ dataset, compare it with existing methods, and conduct a series of ablation experiments. Additionally, we analyze the model’s attention to EEG electrode channels and audio feature attention.

### Comparison with baseline

**Table 2:** Comparison of the performance of EEG and audio data fusion models at different depths of GCN layers

Modality	Method	ACC($\%$)	PRE($\%$)	REC($\%$)	F1 Score($\%$)
Multimodal	MS2-GNN^31^	86.49	82.35	87.50	84.85
	Ahmed et al.^34^	95.78	93.45	95.64	94.53
	Effnetv2s^13^	93.07	92.92	91.76	93.92
	Mobile-Net^13^	83.89	78.81	77.94	78.07
	Hu et al.^35^	80.59	-	-	-
	EMO-GCN	**96.76**	**96.26**	**95.37**	**95.81**
EEG	Tasci et al.^36^	83.96	86.76	76.14	81.10
	SGP-SL^37^	84.91	80.77	87.50	84.00
	Soni et al.^38^	88.80	86.60	87.20	87.10
	Shen et al.^39^	72.25	-	81.88	-
	Sun et al.^40^	84.18	-	78.29	-
	EMO-GCN-$\alpha$	**90.06**	**90.20**	**88.46**	**89.32**
Audio	GNN-SDA^41^	82.70	82.60	79.20	80.90
	Gheorghe et al.^42^	84.16	85.30	83.80	84.00
	Sun et al.^28^	90.35	88.25	90.33	89.15
	Chen et al.^43^	83.40	83.50	76.80	80.00
	Das et al.^44^	90.47	89.53	89.43	89.47
	EMO-GCN-$\beta$	**90.48**	**92.36**	**90.48**	**91.41**

Table 2 presents the comparative experimental results. From these results, it can be observed that EMO-GCN-$\alpha$, which uses EEG data, and EMO-GCN-$\beta$, which uses audio data, achieve accuracy rates of 90.06% and 90.48%, respectively. These rates are approximately 6% lower than the multimodal performance of EMO-GCN, highlighting the value of combining modalities as it enhances the overall accuracy of the model in identifying MDD. Compared to other multimodal methods in the table, EMO-GCN excels in all evaluation metrics, with an accuracy of 96.76%, precision of 96.26%, recall of 95.37%, and F1 score of 95.81%. Although the model proposed by Ahmed et al. achieves a similar accuracy of 95.78%, it falls slightly short in precision and F1 score. Similarly, pretrained models such as Effnetv2s and Mobile-Net reach accuracies of 93.07% and 83.89%, respectively, while the MS2-GNN model and the method by Hu et al. show moderate performance, with accuracies of 86.49% and 80.59%. These results clearly emphasize EMO-GCN’s significant advantage in multimodal data analysis.

In the comparison of EEG unimodal methods, the EMO-GCN-$\alpha$ model demonstrates a strong advantage, achieving the highest scores across all evaluation metrics, with an accuracy of 90.06%, precision of 90.20%, recall of 88.46%, and an F1 score of 89.32%. While other models perform relatively well-such as the model by Soni et al., which achieves an accuracy of 88.80%-their overall performance remains slightly lower than that of EMO-GCN-$\alpha$. Similarly, the SGP-SL model performs well with an accuracy of 84.91%, but its precision and F1 score are lower than those of EMO-GCN-$\alpha$. Other models, such as those proposed by Tasci et al., Shen et al., and Sun et al., achieve relatively lower scores, further underscoring the advantage of EMO-GCN-$\alpha$ in modeling EEG data through graph structures and employing multiple GCNs to learn EEG features.

In the audio modality, EMO-GCN-$\beta$ also outperforms other audio-based models across various metrics, with accuracy, precision, recall, and F1 scores of 90.48%, 92.36%, 90.48%, and 91.41%, respectively. Although the model by Das et al. performs similarly in terms of accuracy and precision, it lags slightly in recall and F1 score. Likewise, the model by Sun et al. achieves an accuracy of 90.35%, but its precision and F1 score are lower than those of EMO-GCN-$\beta$. Other models, including GNN-SDA and the method by Chen et al., exhibit relatively average performance, with all metrics significantly lower than those of EMO-GCN-$\beta$. These results demonstrate the effectiveness of applying GCNs to audio data.

Overall, the performance of EMO-GCN in both multimodal and unimodal experiments confirms its superior capability, making it a powerful model for MDD detection tasks.

### Ablation study

We conducted a detailed ablation study on the network layers of the model to assess the contribution of each part of the model. Given the high dimensionality of EEG signals, we adopted the GraphSAGE technique to achieve dimensionality reduction. After removing the GraphSAGE layer, the model’s ACC dropped to 77.44$\%$, indicating that this technique can effectively reduce the signal dimensions while preserving features critical to differentiating between patients with depression and healthy individuals.

**Table 3:** Comparison of the performance of EEG and audio data fusion models at different depths of GCN layers

Ablation condition	GCN LayerCount	ACC($\%$)	PRE($\%$)	REC($\%$)	F1 Score($\%$)
EEG	4	86.11	85.85	84.26	85.05
	3	**90.06**	**90.20**	**88.46**	**89.32**
	2	84.72	87.50	84.26	85.85
	1	81.94	83.18	82.41	82.79
	0	73.15	76.04	67.59	71.57
Audio	4	62.50	68.32	63.89	66.03
	3	**90.48**	**92.36**	**90.48**	**91.41**
	2	83.80	88.00	84.15	84.62
	1	81.94	83.18	82.41	82.79
	0	73.61	72.64	71.30	71.96

Furthermore, we conducted ablation experiments on the Multi-GCN, as detailed in Table 3. By fixing the GCN layers of one modality and varying the GCN layers of the other, we explored the optimal number of layers for the model. The results show that increasing the GCN layers to 4 in the EEG modality, compared to 3 GCN layers, led to a 4-5$\%$ decrease in all metrics. When the GCN layers were increased to 4 in the audio modality, there was an approximate 20$\%$ decline in all performance metrics, suggesting that overly deep GCN layers might lead to model overfitting. The accuracy of the model also decreased with the reduction of GCN layers in both modalities. It can also be seen from Table 3 that reducing the GCN layers to 2 for each modality resulted in a 6-7$\%$ decrease in all metrics. When the number of GCN layers was reduced to 0, the accuracy metric decreased by about 17$\%$ compared to 3 layers, indicating that fewer GCN layers cannot sufficiently learn signal features. The experimental results confirm the effectiveness of the proposed three-layer Multi-GCN structure.

### EEG electrode channel attention analysis

**Figure 4:**
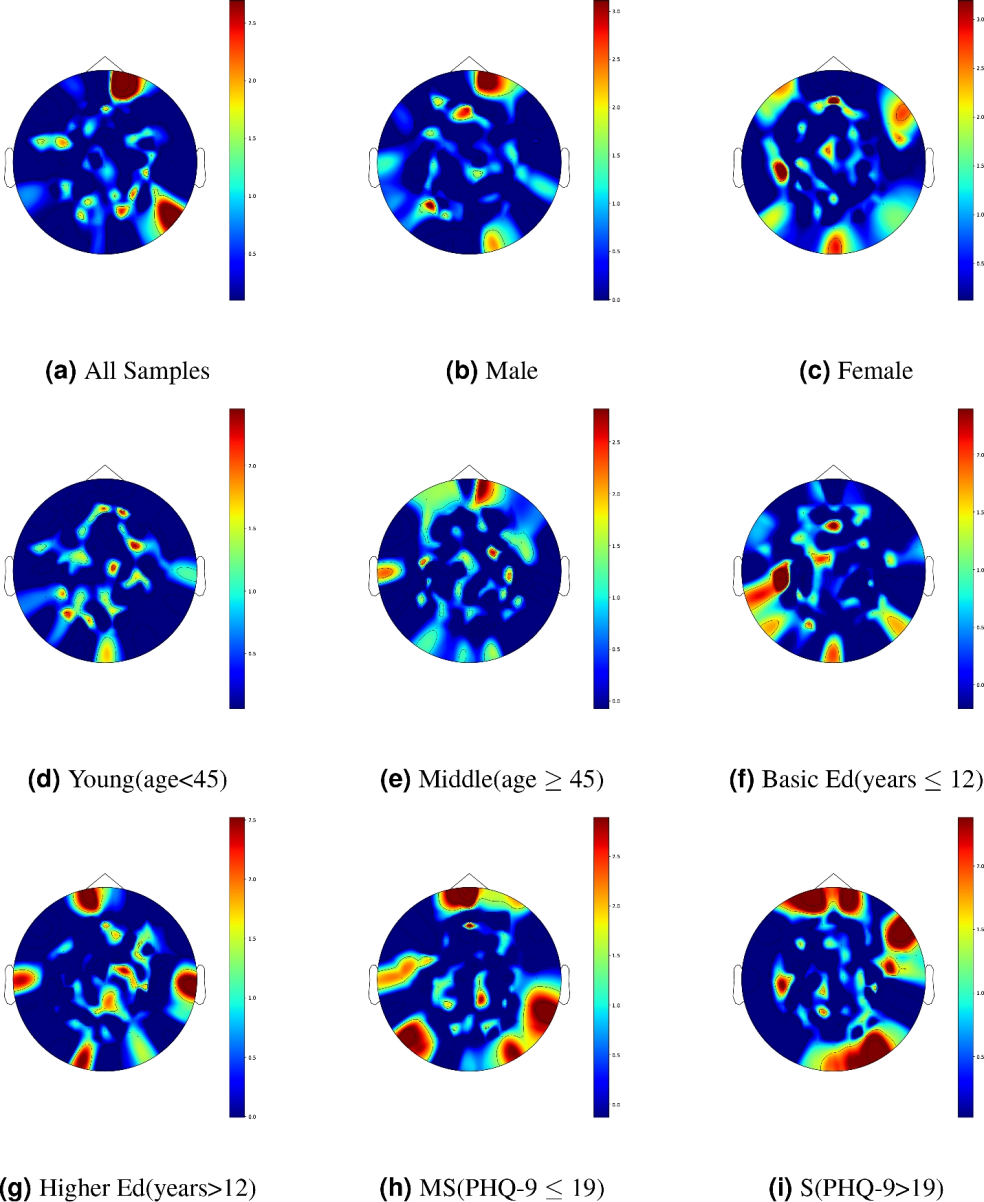
The brain topographic of the attention score.“Ed”, “MS” and “S” denote the education, moderately severe, and severe, respectively.

To investigate the attention of the model proposed in this paper on different EEG electrode channels, we extracted the attention scores for each electrode channel after training the model and mapped these scores onto a brain topography map, resulting in a brain topography Fig. 4a that displays the attention scores for the electrode channels. The areas trending towards red on the chart indicate that the model pays special attention to the electrode channels in the frontal, parietal, and temporal lobes. Moreover, we conducted a categorical analysis based on demographic characteristics and health indicators to explore whether the model’s attention to the electrode channels changes in different contexts. By comparing Fig. 4, we observed significant differences in the brain topography when the model processes data from individuals of different genders, age groups (with 45 years as the threshold), and education levels (with 12 years of education as the threshold). This indicates that the model’s focus shifts when dealing with data from individuals with varying demographic backgrounds, suggesting that there may be differences in the characteristics of depression among different demographic groups. Additionally, we divided the patients with depression into moderately severe and severe groups based on their PHQ-9 scores and conducted studies, finding that the model’s focus on electrode channels also differs between patients with moderate and severe depression.

In our study, the areas of high attention in the Fig. 4a largely coincide with regions that were found to differ in degree distribution brain topography between MDD patients and healthy subjects in the research by Li et al.^46^ This finding suggests that the neural network model we proposed can automatically identify differences between MDD patients and healthy individuals in EEG data, and significantly focus on these regions of pronounced differences.

### Audio feature attention analysis

**Figure 5:**
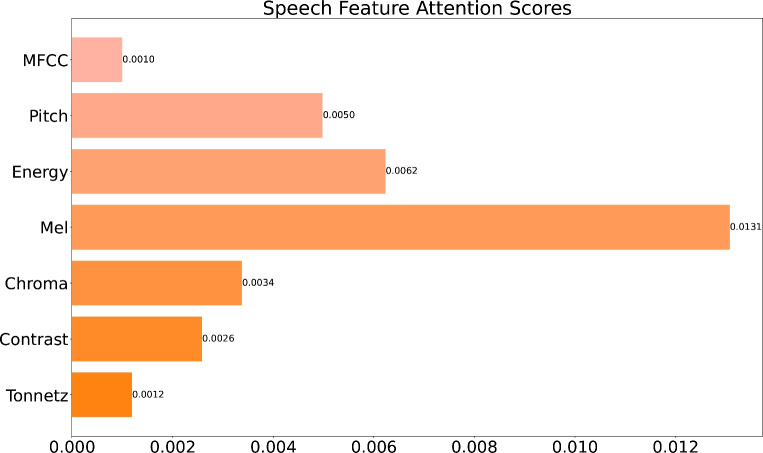
Speech feature attention rank.Here we apply a linear transformation to the attention score, taking the smallest attention score as the baseline value and subtracting the baseline value

In this study, the developed model identifies signs of depression by analyzing audio features. To assess the importance of different audio features, the model assigns attention scores to seven features-MFCC, Pitch, Energy, Mel-Spectrum, Chroma, Contrast, and Tonnetz. These attention scores offer a quantified means to evaluate the contribution of each feature to the model’s ability to recognize depression.

As shown in Fig. 5, the Mel feature obtained the highest attention scores, indicating that the model considers the Mel to be the most important among all the considered sound features for recognizing depression. By simulating the auditory perception characteristics of the human ear, the Mel can effectively capture the perceptual content of sounds, making it particularly useful in identifying emotional and mental states. This result proves that the Mel plays a key role in analyzing emotion-related sound changes. Pitch and energy features also received relatively high attention scores, suggesting an association between these fundamental acoustic features and emotional expression. In contrast, MFCC, Chroma, Contrast, and Tonnetz features received lower attention scores, indicating that in the specific context and dataset of this study, these features contribute le ss to distinguishing depressive states. However, this does not mean that these features are irrelevant in emotional analysis; rather, their direct impact on identifying depression is less significant within the framework of this study.

## Discussion

In this study, we developed an innovative method for depression recognition, which relies on a graph neural network architecture that combines graph convolutional layers, graph pooling operations, and structural learning to extract key features from multimodal data and effectively integrate these features through an attention mechanism. We named this method EMO-GNN and conducted a detailed comparative analysis with existing depression detection methods. The results show that our method consistently demonstrates superior performance across various scenarios, particularly in multimodal contexts, where EMO-GCN emerged as the most outstanding model. Furthermore, through a series of ablation experiments, we validated the importance of each network layer in the Multi-GCN component of our model. The results clearly showed a significant decline in model performance with the addition or removal of GCN layers, thereby confirming the effectiveness of our proposed three-layer GCN structure. Lastly, we conducted an in-depth analysis of the model’s attention to different EEG electrode channels and various acoustic features. The results are consistent with existing research, further validating that EMO-GCN can reveal complex relationships within the data.

Our study still faces several limitations. On the data front, like many others, we resorted to segmenting data to expand the dataset, primarily due to the current scarcity of depression-related data. Additionally, we aim to integrate more types of modal data, allowing for a more comprehensive perspective in diagnosing depression. In terms of clinical validation and acceptance, while our initial results show potential effectiveness, our research has yet to undergo extensive clinical trials. Furthermore, the interpretability of the model is a key factor in its adoption by medical professionals. Deep learning models are often criticized as “black box” models, which poses a significant challenge in the medical field that demands high transparency and interpretability. In the future, we hope to access more EEG and audio data from MDD patients, as well as data from other modalities, to explore the diversity and variability across different datasets more deeply. Additionally, we aim to enhance the model’s interpretability further, enabling medical professionals and patients to better understand the decision-making process of the model.

## Conclusion

In this paper, we introduce EMO-GCN, a multimodal depression detection framework based on graph neural networks. We consider a graph convolutional layer and a graph pooling layer together as a fundamental unit. By stacking such units threefold, we construct the core component of EMO-GCN, Multi-GCN. This configuration enables the model to extract key features from different modalities and integrate these features through an attention mechanism, achieving high-accuracy diagnosis of depression and exploring the potential connections between EEG data and audio data.

Through a series of rigorous experimental validations, our method surpassed the performance of all currently known methods in multimodal depression detection. The experimental analysis indicated that the GraphSAGE component within the model, as well as the graph convolutional layers and graph pooling operations in the graph neural network, played significant roles in enhancing model performance. Moreover, the model also demonstrated high accuracy when processing unimodal data, and the integration of multimodal features further increased detection precision. These results not only confirm the effectiveness of EMO-GCN in the field of depression detection but also highlight the potential of multimodal data fusion in improving diagnostic accuracy.

## Data Availability

This study used the publicly available MODMA dataset, and these data can be obtained directly from the corresponding official website https://modma.lzu.edu.cn/data/index/.
